# End of life in the critically ill patient: evaluation of experience of end of life by caregivers (EOLE study)

**DOI:** 10.1186/s13613-021-00944-z

**Published:** 2021-11-26

**Authors:** The SRLF Trial Group, Nadia Aissaoui, Virginie Amilien, Nadiejda Antier, Adrien Auvet, Elie Azoulay, Saber Davide Barbar, Florent Bavozet, Asael Berger, Sami Blidi, Florence Boissier, Pierre Bouju, Yannick Brunin, Bertrand Canoville, Maguelone Chalies, Frank Chemouni, David Couret, Marc Danguy, Cédric Daubin, Guillaume Decormeille, Alexandre Demoule, Julien Duvivier, Stephan Ehrmann, Etienne Escudier, Pierre Esnault, Arnaud Galbois, Mathieu Guilbart, David Grimaldi, Nicholas Heming, Alexandre Herbland, Bertrand Hermann, Clément Hoffmann, Stéphanie Houcke, Sami Hraeich, Frédéric Jacobs, Gwenaelle Jacq, Amira Jamoussi, Sébastien Jochmans, Nancy Kentish-Barnes, Jean-Claude Lacherade, Fabien Lambiotte, Jean-Baptiste Lascarrou, Gabriel Lejour, Jean-François Llitjos, Cécile Lory, Guillaume Louis, Estelle Martin, Philippe Mateu, Jonathan Messika, Philippe Michel, Jean-Paul Mira, Sébastien Moschietto, Grégoire Muller, Lamia Ouanes-Besbes, François Philippart, Michael Piagnerelli, Gael Piton, Gaetan Plantefeve, Laurent Poiroux, Jean-Pierre Quenot, Jean Reignier, Anne Renault, René Robert, Arnaud Sement, Pierre-Yvan Simonoviez, Anne Terrier, Martial Thyrault, Jean Turc, Thierry Vanderlinden, Atika Youssoufa

**Affiliations:** Paris, France

**Keywords:** End of life, Withdrawal treatment, Withholding treatment, Critical care

## Abstract

**Background:**

The death rate in intensive care units (ICUs) can reach 20%. More than half occurs after a decision of care withholding/withdrawal. We aimed at describing and evaluating the experience of ICU physicians and nurses involved in the end-of-life (EOL) procedure. Primary objective was the evaluation of the experience of EOL assessed by the CAESAR questionnaire. Secondary objectives were to describe factors associated with a low or high score and to examine the association between Numeric Analogic Scale and quality of EOL.

**Methods:**

Consecutive adult patients deceased in 52 ICUs were included between April and June 2018. Characteristics of patients and caregivers, therapeutics and care involved after withdrawal were recorded. CAESAR score included 15 items, rated from 1 (traumatic experience) to 5 (comforting experience). The sum was rated from 15 to 75 (the highest, the best experience). Numeric Analogic Scale was rated from 0 (worst EOL) to 10 (optimal EOL).

**Results:**

Five hundred and ten patients were included, 403 underwent decision of care withholding/withdrawal, and among them 362 underwent effective care withdrawal. Among the 510 patients, mean CAESAR score was 55/75 (± 6) for nurses and 62/75 (± 5) for physicians (*P* < 0.001). Mean Numeric Analogic Scale was 8 (± 2) for nurses and 8 (± 2) for physicians (*P* = 0.06). CAESAR score and Numeric Analogic Scale were significantly but weakly correlated. They were significantly higher for both nurses and physicians if the patient died after a decision of withholding/withdrawal. In multivariable analysis, among the 362 patients with effective care withdrawal, disagreement on the intensity of life support between caregivers, non-invasive ventilation and monitoring and blood tests the day of death were associated with lower score for nurses. For physicians, cardiopulmonary resuscitation the day of death was associated with lower score in multivariable analysis.

**Conclusion:**

Experience of EOL was better in patients with withholding/withdrawal decision as compared to those without. Our results suggest that improvement of nurses’ participation in the end-of-life process, as well as less invasive care, would probably improve the experience of EOL for both nurses and physicians.

*Registration*: ClinicalTrial.gov: NCT03392857.

**Supplementary Information:**

The online version contains supplementary material available at 10.1186/s13613-021-00944-z.

## Background

End of life (EOL) is a frequent event in intensive care units. Variations in type, frequency, and timing of end-of-life decisions were observed in a large international study. Withholding treatment was more common than withdrawing treatment [1]. Many improvements can still be done with end-of-life care worldwide, still raising ethical, legal, political, psychological and medical questions [2]. It requires specific and particular care from caregivers (for the patient as well as for the relatives). Notwithstanding the frequency of these situations, caregivers may still be uncomfortable with the dying patient [3]. Some studies have evaluated caregivers’ perception of death in ICU [4–8]*,* most of them using the QODD scale (Quality Of Dying and Death in the ICU)*.* Nurses rating varied between countries [6]. A 15-item questionnaire named CAESAR was recently developed and validated to assess the experience of relatives [9] and caregivers [10]. In the first study, relatives’ lower scores were associated with greater risks of anxiety and depression at 3 months, of post-traumatic stress-related symptoms at 3, 6 and 12 months and of complicated grief at 6 and 12 months [9], a finding emphasizing the importance of a good experience of end-of-life. The second study showed important areas for improving practices, including appropriate symptom control and goals of care, quality teamwork, quality communication and involvement of family members [10]. The aim of our study was to describe the experience of end-of-life by caregivers using the new CAESAR questionnaire and to describe the factors associated with a low or high score.

## Methods

### Design

We conducted a prospective multicenter observational study between April and June 2018 in 52 ICUs. According to the number of patients annually admitted in each ICU, 5 to 15 consecutive adult patients deceased in ICUs were included (5 patients for centers with < 400 annual admissions, 10 patients for centers with annual admissions between 400 and 800 and 15 patients for centers with annual admission > 800).

### Ethics

The study was approved by the institutional review board of “Comité de Protection des Personnes Ile de France III”, and was registered on Clinical Trial as NCT03392857. Need for informed consent was waived due to the study’s observational design and in accordance with the French law. This study was conducted in accordance with the principles of the Declaration of Helsinki. Relatives of the patients and caregivers were informed of the study and gave their non-opposition to participation.

### Objectives

The primary objective was to evaluate the experience of end of life for ICU nurses and physicians using the CAESAR questionnaire assessing burden of the experience of death and dying in an ICU. CAESAR is a 15-item questionnaire, previously validated in a multicenter prospective study in French ICUs [10]. Each item included a written description and a score on a 5-point scale (1, traumatic; 2, painful; 3, difficult; 4, acceptable; 5, comforting). The sum total of the items was rated from 15 to 75 (the higher, the better the experience). The secondary objectives of the study were to describe factors associated with a low or high score, and to examine the correlation between CAESAR score and Numeric Analogic Scale.

### Eligibility

In each ICU, intensivists included consecutive adults who died in the ICU, excluding patients with brain death, and refusal of the next-of-kin.

### Data collection

For each patient, the physician and the nurse in charge of the patient at the time of death (even if they were taking care of the patient for only one or a few days) were asked to complete the CAESAR questionnaire within 24 h. We also evaluated the experience of end of life with a Numeric Analogic Scale (10-point Likert scale) rated from 0 (traumatic) to 10 (optimal), as in other studies [11], in order to have a global evaluation of this experience, and in order to compare it to the CAESAR evaluation.

We also collected data concerning characteristics of ICUs, caregivers, patients and end-of-life process, such as use of vasoactive drugs, renal replacement therapy, invasive or non-invasive ventilation, cardiopulmonary resuscitation, monitoring, introduction and management of sedation by hypnotics and/or morphine derivate. We also collected information on whether the patient died after a decision of withholding/withdrawal, in order to compare the experience of end of life with and without such a decision.

### Statistical analysis

Qualitative variables were described as n (%) and quantitative variables as mean ± SD if normally distributed and median [25th–75th percentiles] otherwise. Qualitative variables were compared across groups using the Chi-square test or Fisher’s exact test as appropriate. To compare quantitative variables across groups, we applied Student’s *t* test or the Wilcoxon Mann–-Whitney test.

We also built two multiple linear regression models to identify factors associated with CAESAR score after effective care withdrawal: first for nurses and second for physicians, with random-effect multilevel logistic regression to take into account the effect of the center. The analyses were adjusted for potential confounders defined as factors associated with CAESAR score at *P* values ≤ 0.2 by univariate analysis.

No imputation for missing data was carried out and no adjustments were made for the multiple comparisons.

All statistical tests were two-sided and *P* values of 0.05 or less were considered significant. All statistical analyses were performed using Python® (Python Software Foundation, https://www.python.org/).

## Results

### Characteristics of centers, patients and caregivers

Four hundred and fifty-five caregivers (258 nurses and 197 physicians) of 510 deceased patients were included in 52 ICUs (representing 27% of the solicited centers). ICUs were mainly mixed (receiving both post-operative and medical patients) ICUs (67%), with a mean annual admission number of 849 (± 525). Thirty-four ICUs (65%) had regular meetings to discuss ethical issues. A psychologist was available for caregivers in 63% of ICUs, and 62% of the units had the possibility of post-death debriefing meetings for caregivers. Patients had a median age of 70 [61–79] years. Main reasons of admission were acute respiratory failure (32%), shock (24%), cardiac arrest (19%), and coma (12%). Other characteristics of patients are described in Table 1. Patients without decision of care withholding/withdrawal had a lower McCabe score, and more support during the ICU stay, and the day of death (vasoactive drugs, invasive ventilation, renal replacement therapy, extra-corporeal life support) (Table 1). Characteristics of caregivers are described in Table 2.

**Table 1:** Characteristics of patients

	All patients		Patients with decision of care withholding /withdrawal		Patients without decision of care withholding/withdrawal		*P*
Patients	N 510	Median [IQR]	N 403	Median [IQR]	N 107	Median [IQR]	
Age, years	509	70 [61–79]	402	71 [61–79]	107	67 [59–76]	0.06
SAPS 2	502	65 [50–80]	398	62 [50–76]	104	75 [55–97]	0.33
McCabe score	506	2 [1–3]	400	2 [1–3]	106	2 [1–3]	< 0.01
Delay between admission and decision of withdrawal/withholding, days	375	6 [2–12]	375	6 [2–12]			
Delay between decision of withdrawal and death, days	358	0 [0–1]	358	0 [0–1]			

During the stay in ICU		N (%)					
Vasoactive drugs	510	390 (76%)	403	287 (71%)	107	103.0 (96%)	< 0.01
Invasive ventilation	510	435 (85%)	403	334 (83%)	107	101 (94%)	< 0.01
Non-invasive ventilation	508	143 (28%)	402	115 (29%)	106	28 (26%)	0.75
Renal replacement therapy	510	143 (28%)	403	90 (22%)	107	53 (49%)	< 0.01
Extra-corporeal life support	509	22 (4%)	403	11 (3%)	106	11 (10%)	< 0.01

The day of death		N (%)					
Vasoactive drugs	510	256 (50%)	403	157 (39%)	107	99 (93%)	< 0.01
Invasive ventilation	509	361 (71%)	402	265 (66%)	107	96 (90%)	< 0.01
Non-invasive ventilation	508	50 (10%)	402	45 (11%)	107	5 (5%)	0.07
Renal replacement therapy	501	76 (15%)	394	36 (9%)	107	40 (37%)	< 0.01
Extra-corporeal life support	509	21 (4%)	403	9 (2%)	106	12 (11%)	< 0.01

**Table 2:** Characteristics of caregivers

Caregivers	Physicians	Nurses
Age, years, median [IQR]	37 [33–47] *N* = 197	30 [26–36] *N* = 258
Number of years in the unit, median [IQR]	7 [4–15] *N* = 197	6 [3–10] *N* = 258
Religious belief	49.6% *N* = 197	45.8% *N* = 258
Taking care of the patient for one or a few days	81% *N* = 496	89% *N* = 480
Disagreements between caregivers on the intensity of life support	9.1% *N* = 496	12.3% *N* = 480
Disagreements between caregivers and relatives	7.7% *N* = 496	7.1% *N* = 476
Disagreements between ICU caregivers and other department’s physicians	5.1% *N* = 493	3.0% *N* = 474

Four hundred and three patients (79%) underwent decision of care withholding/withdrawal, and among them, 362 underwent effective life support withdrawal. The decision was mainly taken during the daily meeting (34%) or during a dedicated meeting (34%). In 74% of cases, a nurse was present, and in this case, participated actively in the discussion in 72% of cases. The physician took the decision alone in 4% of cases, or with another physician during an informal meeting in 24% of cases (other cases or lack of information: 4%). First reason for withholding/withdrawal was the certainty of short-term death (75% of patients). The main other reasons were dependence for activity of daily living before hospitalization (21% of patients), the certainty of dependence after hospitalization (40% of patients), and advanced age in 19% (multiple answers were possible). Among the 362 patients with effective life support withdrawal, 12 had a cardiopulmonary resuscitation the day of death (11 before the decision of support withdrawal, and 1 after).

Among the 362 patients with effective life support withdrawal, 278 (77%) received sedation with benzodiazepine, 61 received another sedation (17%), and 318 (88%) received opioids.

Reasons for initiating sedation were to relieve pain in 19% of cases, to relieve dyspnea and anxiety in 37 and 22%, respectively, bronchial congestion in 25%, gasps in 18%, and in response to a request from the relatives or the paramedical team in 28% of cases (multiple answers were possible). Time lapse to death was not significantly different whether patients received sedation or not (*P* = 0.17).

### CAESAR score and Numeric Analogic Scale

**Fig. 1:**
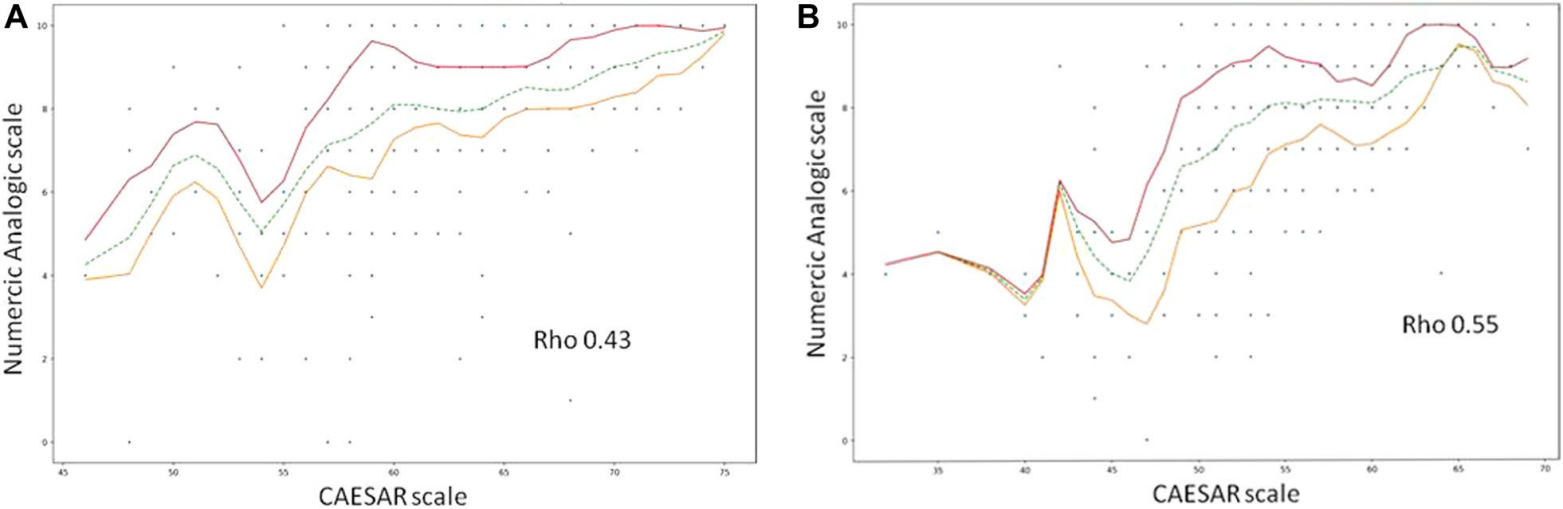
**A** Correlation between CAESAR score and Numeric Analogic Scale for physicians. **B** Correlation between CAESAR score and Numeric Analogic Scale for nurses

Three hundred and eighty-seven CAESAR scores were fully completed by 197 physicians and 312 scores by 258 nurses (Table 3: score and number of answers per item; Additional file 1: score per item for patients with decision of care withholding/withdrawal; Additional file 2: score per item for patients without decision of care withholding/withdrawal). Mean CAESAR score was significantly lower for nurses (55 ± 6) than for physicians (62 ± 5; *P* < 0.001) (median score for nurses 56 [52–59], and for physicians 62 [59–66]). 502 Numeric Analogic Scales were completed by physicians and 479 by nurses. Mean Numeric Analogic Scale was 8 (± 2) for nurses and 8 (± 2) for physicians (P = 0.06). CAESAR score and Numeric Analogic Scale for both nurses and physicians were significantly higher if the patient died after a decision of withholding/withdrawal (Table 4). Correlation between CAESAR and Numeric Analogic Scale was weak (*P* < 0.001 but Pearson’s r of 0.43 for physicians and of 0.55 for nurses) (Fig. 1A and B).

**Table 3:** Physician and nurse CAESAR scores (EOL = end-of-life)

	Physician Mean score (± SD)	Nurse Mean score (± SD)
Items for physicians and nurses		
**1. Was an EOL palliative care approach clearly decided for the patient?** Please rate this experience: 1 Traumatic. 2 Painful. 3 Difficult. 4 Acceptable. 5 Comforting	4.25 (± 0.58) *N* = 483	4.06 (± 0.65) *N* = 464
**2. Was the decision to withhold or withdraw treatment clearly documented in the medical report?** Please rate this experience: 1 Traumatic. 2 Painful. 3 Difficult. 4 Acceptable. 5 Comforting	4.29 (± 0.57) *N* = 423	4.05 (± 0.65) *N* = 362
**3. Do you think the patient received excessive or futile care?** Please rate this experience: 1 Traumatic. 2 Painful. 3 Difficult. 4 Acceptable. 5 Comforting	4.17 (± 0.59) *N* = 488	3.85 (± 0.62) *N* = 467
**4. Was the patient able to communicate with you during his/her ICU stay?** Please rate this experience: 1 Traumatic. 2 Painful. 3 Difficult. 4 Acceptable. 5 Comforting	3.94 (± 0.58) *N* = 484	4.26 (± 0.7) *N* = 470
**5. Was the patient’s pain under control?** Please rate this experience: 1 Traumatic. 2 Painful. 3 Difficult. 4 Acceptable. 5 Comforting	4.23 (± 0.56) *N = *480	3.77 (± 0.77) *N* = 466
**6. Was the patient able to breathe comfortably?** Please rate this experience: 1 Traumatic. 2 Painful. 3 Difficult. 4 Acceptable. 5 Comforting	3.94 (± 0.62) *N* = 486	4.23 (± 0.66) *N* = 473
**7. In your opinion. was the patient’s dignity respected?** Please rate this experience: 1 Traumatic. 2 Painful. 3 Difficult. 4 Acceptable. 5 Comforting	4.31 (± 0.57) *N* = 489	4.18 (± 0.76) *N* = 464
**8. Did the relatives pay regular visits to the patient?** Please rate this experience: 1 Traumatic. 2 Painful. 3 Difficult. 4 Acceptable. 5 Comforting	4.27 (± 0.65) *N* = 490	4.21 (± 0.78) *N* = 451
**9. Did the ICU team discuss the patient’s EOL wishes with the patient him/herself or with the relatives?** Please rate this experience: 1 Traumatic. 2 Painful. 3 Difficult. 4 Acceptable. 5 Comforting	4.22 (± 0.79) *N* = 487	3.86 (± 0.83) *N* = 449
**10. Were the relatives at the patient’s bedside at the time of death?** Please rate this experience: 1 Traumatic. 2 Painful. 3 Difficult. 4 Acceptable. 5 Comforting	4.09 (± 0.59) *N* = 487	4.02 (± 0.91) *N* = 468
**11. During the patient’s ICU stay. did the relatives receive support from a psychologist?** Please rate this experience: 1 Traumatic. 2 Painful. 3 Difficult. 4 Acceptable. 5 Comforting	4.19 (± 0.72) *N* = 488	3.84 (± 0.72) *N* = 475
**12. Are you satisfied with the patient’s overall quality of dying and death?** Please rate this experience: 1 Traumatic. 2 Painful. 3 Difficult. 4 Acceptable. 5 Comforting	3.75 (± 0.67) *N* = 484	3.74 (± 0.78) *N* = 441
**13. If the patient had been your relative. would you have been satisfied with his/her EOL?** Please rate this experience: 1 Traumatic. 2 Painful. 3 Difficult. 4 Acceptable. 5 Comforting	4.09 (± 0.56) *N* = 489	4.0 (± 0.71) *N* = 474
**Specific physician items**		
**14. Were the relatives able to say good-bye to the patient?** Please rate this experience: 1 Traumatic. 2 Painful. 3 Difficult. 4 Acceptable. 5 Comforting	4.3 (± 0.62) *N* = 489	
**15. Did you experience conflict with the patient and/or the relatives?** Please rate this experience: 1 Traumatic. 2 Painful. 3 Difficult. 4 Acceptable. 5 Comforting	3.86 (± 0.93) *N* = 488	
**Specific nurse items**		
**14. Were the relatives able to have physical contact (touch. hug) with the patient?** Please rate this experience: 1 Traumatic. 2 Painful. 3 Difficult. 4 Acceptable. 5 Comforting	4.08 (± 0.7) *N* = 471	
**15. Were you present at the patient’s bedside at the time of death?** Please rate this experience: 1 Traumatic. 2 Painful. 3 Difficult. 4 Acceptable. 5 Comforting	3.39 (± 1.13) *N* = 459

**Table 4:** CAESAR score and Numeric Analogic Score

	Global score Mean ± SD	Patients with withholding/withdrawal decision (N = 403) Mean ± SD	Patients without withholding/withdrawal decision (N = 107) Mean ± SD	*P*
Physicians’ CAESAR Score (*N* = 387)	62 ± 5	62 ± 5	61 ± 5	0.002
Nurses’ CAESAR Score (*N* = 312)	55 ± 6	56 ± 6	54 ± 6	< 0.001
Physicians’ Numeric Analogic Scale (*N* = 502)	8 ± 2	8 ± 2	7 ± 2	0.03
Nurses’ Numeric Analogic Scale (*N* = 479)	8 ± 2	8 ± 2	7 ± 3	< 0.001

### Factors associated with CAESAR score

In univariable analysis, for physicians and nurses, renal replacement therapy (*P* = 0.03, *P* < 0.001, respectively), and cardiopulmonary resuscitation (*P* < 0.001, *P* = 0.01, respectively) the day of death were associated with significantly lower CAESAR score. For nurses, variables associated with a significantly lower CAESAR score were: invasive ventilation during the stay in ICU (*P* < 0.001), non-invasive ventilation the day of death (*P* = 0.02), and disagreements on the intensity of life support (between caregivers *P* < 0.001; between caregivers and relatives *P* = 0.05; and between ICU caregivers and other department’s physicians *P* = 0.02). For physicians, the presence of a nurse during the decision meeting was associated with a higher score (*P* = 0.02) (Additional file 3).

In univariable analysis, among the 362 patients with effective life support withdrawal, immediate extubation for nurses (*P* = 0.04), and, for nurses and physicians, and discontinuation of blood tests, radiographies, and monitoring (respectively, *P* = 0.02 and *P* = 0.04) were associated with a significant higher CAESAR score (Additional file 3).

**Fig. 2:**
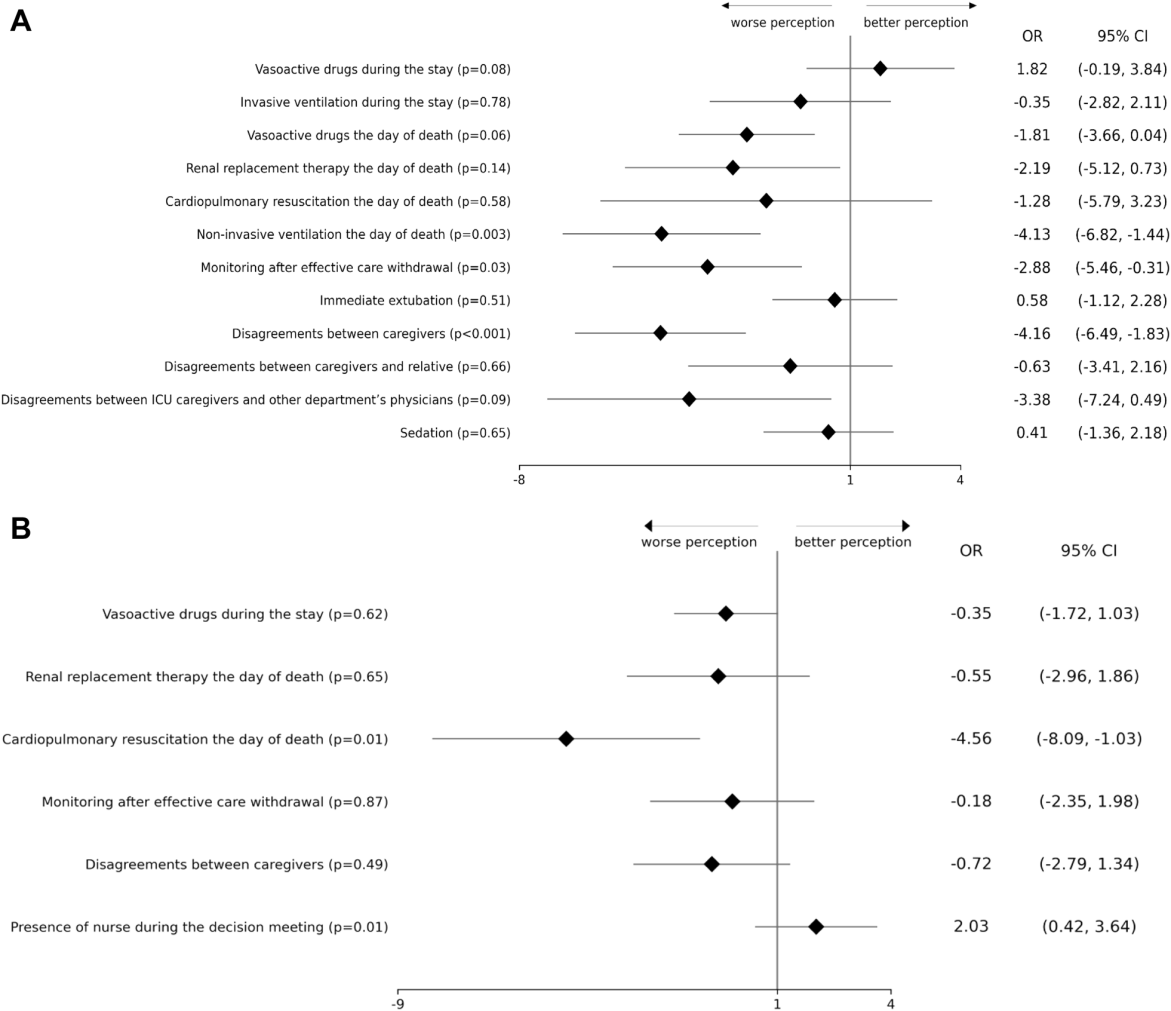
**A** Factors associated with nurses’ CAESAR score in multivariable analysis with random-effect multilevel logistic regression (Forest plot). **B** Factors associated with physicians’ CAESAR score in multivariable analysis with random-effect multilevel logistic regression (Forest plot)

In multivariable analysis with random-effect multilevel logistic regressions, taking into account the effect of the centers, among the 362 patients with effective care withdrawal, disagreement on the intensity of life support between caregivers, as well as non-invasive ventilation the day of death and monitoring and blood tests the day of death were associated with lower CAESAR score for nurses (Fig. 2A). For physicians, cardiopulmonary resuscitation the day of death was associated with lower CAESAR score (Fig. 2B).

## Discussion

The evaluation of the experience of end of life assessed by the CAESAR questionnaire in this multicenter study showed higher scores for physicians (62 ± 5) than for nurses (55 ± 6). CAESAR score for both nurses and physicians was significantly higher if the patient died after a decision of withholding/withdrawing treatment.

The global score was higher (better) for physicians than for nurses. The nurses’ and the physicians’ CAESAR questionnaire cannot however be strictly compared, as 2 questions differed between the questionnaires, but previous studies have shown that nurses and residents report lower QODD scores than families and physicians [8].

The scale used in previous studies [5, 6, 8] was mainly QODD, which was designed and validated in United States. The study by Gerristen et al. showed differences between QODD rating by nurses in United States and Netherlands, which might be due to cultural or organizational differences, or differences in perceptions and expectations [6].

The CAESAR questionnaire was created and validated in France. It would be interesting in further studies to compare both QODD and CAESAR scores in the same caregiver population.

In the previous study validating and evaluating the CAESAR scale, the median global CAESAR score was higher for nurses (62/75 [59–66] versus 56/75 [52–59] in our study), and for physicians (64/75 [61–68] versus 62/75 [59–66] in our study). A score between 45 and 60 is considered intermediary, showing that some elements of the end-of-life were experienced negatively [10].

In the CAESAR study, intensivists included consecutive adults who died after at least 48 h in the ICU and who received at least one visit from their relatives, two factors that may change perception of EOL for the caregiver. Severity of patients was also higher in our study: median SAPS 2 was 65 (50–80) versus 58 (44–71) in CAESAR study, with patients more frequently ventilated (85% versus 71%) [10]. These differences, mainly in the nurse score, could also be explained by a selection bias of centers, which belong to the FAMIREA study group in the first study, and are perhaps more involved in the evaluation of the end-of-life accompaniment [10]. In our study, only 19 centers (36%) participated in recent studies ARREVE and CAESAR [9, 11].

In France, nurses’ perception of death was evaluated some years ago in hospitals [12] and in ICUs [4], showing poor estimation of quality of death [12] and of end-of-life decision, but since then French law has changed (in 2005 and in 2016), allowing withdrawal, withholding, and palliative sedation.

In a recent multicenter French study [13], nurses rated the end of life of their patients under mechanical ventilation at 8 on a scale from 1 (worst) to 10 (best), concordant with our findings of Mean Numeric Analogic Scale of 8 (± 2) for nurses.

In multivariable analysis, disagreement on the intensity of life support between caregivers was associated with lower CAESAR score for nurses.

Disagreement with physicians was also reported in the study of validation of the CAESAR score for nurses [10], and end-of-life care was one of the main reported sources of conflict in a previous European study about intensive care conflicts [14]. Improvement of nurses’ participation in the end-of-life process would probably improve their experience of end-of-life and might decrease job-related symptoms. It could also improve the experience of physicians, as suggested by our result showing that the presence of nurses in the decision meeting was associated with a higher score in univariable analysis.

In our study, for both nurses and physicians, experience of good perception of death was associated with an absence of life-sustaining therapies (probably considered as excessive and futile in this setting). These findings are concordant with other studies [7, 10]. In our study, among 362 patients with effective life support withdrawal, 12 had cardiopulmonary resuscitation the day of death (11 before the decision of support withdrawal and 1 after), a finding probably reflecting excessive treatment in patients with poor prognosis. It underscores the need to determine objectives about end of life with patients and caregivers, when possible, and to anticipate advance palliative care planning [15].

The number of patients undergoing effective care withdrawal is high in our study (70% of the patients included in the study, which means 70% of patients dying in the ICU). In the CAESAR study, only 19% of dying patients included in the study had made no decision to withhold or withdraw life-sustaining therapies, and 48% had made a withdrawal decision [9]. In the Ethicus 2 study, treatment withdrawal was observed in 52.8% of included patients in Northern Europe, but the denominator was not the same, including not only patients who died, but also those who had a limitation of life-sustaining treatment without dying in the ICU [1]. The high rate of withdrawal in our study could be explained by the high McCabe score (median 2 [1–3]) and the severity of patients [median SAPS 2:65 (50–80)].

Our study also evaluated end-of-life perception after a new law in France specifically allowing palliative sedation for end-of-life patients (Claeys-Leonetti law, 2016). Our results showed that among the 362 patients with effective life support withdrawal, 77% received sedation with benzodiazepine, and 88% received opioids. Time lapse to death was not significantly different whether or not patients received sedation, in keeping with a recent study by Robert et al. that showed no relationship between dosages of midazolam or morphine and time before death [13].

The strength of our study lies in its being a large-scale multicentre prospective observational study. Almost two-thirds of the centers had a psychologist available, and post-death debriefing meetings for caregivers were organized. Our study identified modifiable factors associated with end-of-life experience for physicians and nurses. The results suggest that less invasive care, less monitoring, and prevention and resolution of disagreements on the intensity of life support could improve caregivers’ experience of end-of-life.

This study has several limitations. First, almost all participating ICUs were in France. We cannot know whether end-of-life perception evaluation in other countries would have the same results. Second, the global CAESAR score was high and the differences in score were small, and may not be meaningful for clinicians. Third, CAESAR questionnaire was mainly evaluated for end-of-life with withdrawal or withholding treatments, and one of the questions in the CAESAR questionnaire (Was the decision to withdraw or withhold treatments clearly reported in the medical report?) was difficult to answer in case of patients dying without a withdrawal or withholding decision. Fourth, we did not evaluate the impact of long-term exposure of caregivers.

Finally, caregivers’ experience is a key point of the end-of-life process, but while it may be, as is the relatives’ experience, a surrogate for the patient’s experience, it may in some cases represent a self-fulfilling prophecy.

## Conclusion

In conclusion, experience of end-of-life was better in patients with a decision to withhold/withdraw treatment as compared to those without. Our results suggest that improvement of nurses’ participation in the end-of-life process, as well as less invasive care, would probably improve the experience of EOL for both nurses and physicians.

## Supplementary Information

**Additional file 1.** Physician and Nurse CAESAR scores for patients with decision of carewithholding/withdrawal (N = 403).

**Additional file 2.** Physician and Nurse CAESAR scores for patients without decision of carewithholding/withdrawal (N = 107).

**Additional file 3.** Factors associated with CAESAR score (univariable analysis, comparisonbetween presence and absence of each variable, p significant when ≤ 0.05).

## Data Availability

The datasets used and/or analyzed during the current study are available from the secretary of SRLF on reasonable demand (secretariat@srlf.org).

## Abbreviation

EOL: End of life
