# Correction to: End of life in the critically ill patient: evaluation of experience of end of life by caregivers (EOLE study)

**DOI:** 10.1186/s13613-021-00973-8

**Published:** 2021-12-29

**Authors:** The SRLF Trial Group, Nadia Aissaoui, Virginie Amilien, Nadiejda Antier, Adrien Auvet, Elie Azoulay, Saber Davide Barbar, Florent Bavozet, Asael Berger, Sami Blidi, Florence Boissier, Pierre Bouju, Yannick Brunin, Bertrand Canoville, Maguelone Chalies, Frank Chemouni, David Couret, Marc Danguy, Cédric Daubin, Guillaume Decormeille, Alexandre Demoule, Julien Duvivier, Stephan Ehrmann, Etienne Escudier, Pierre Esnault, Arnaud Galbois, Mathieu Guilbart, David Grimaldi, Nicholas Heming, Alexandre Herbland, Bertrand Hermann, Clément Hoffmann, Stéphanie Houcke, Sami Hraeich, Frédéric Jacobs, Gwenaelle Jacq, Amira Jamoussi, Sébastien Jochmans, Nancy Kentish-Barnes, Jean-Claude Lacherade, Fabien Lambiotte, Jean-Baptiste Lascarrou, Gabriel Lejour, Jean-François Llitjos, Cécile Lory, Guillaume Louis, Estelle Martin, Philippe Mateu, Jonathan Messika, Philippe Michel, Jean-Paul Mira, Sébastien Moschietto, Grégoire Muller, Lamia Ouanes-Besbes, François Philippart, Michael Piagnerelli, Gael Piton, Gaetan Plantefeve, Laurent Poiroux, Jean-Pierre Quenot, Jean Reignier, Anne Renault, René Robert, Arnaud Sement, Pierre-Yvan Simonoviez, Anne Terrier, Martial Thyrault, Jean Turc, Thierry Vanderlinden, Atika Youssoufa

**Affiliations:** Paris, France

## Correction to: Annals of Intensive Care (2021) 11:162 https://doi.org/10.1186/s13613-021-00944-z

In the original publication of the article [1], one of the author’s name was published incorrectly as Michael Piagnarelli instead of Michael Piagnerelli. The original article has been corrected.
