# Supplementary material for: End of life in the critically ill patient: evaluation of experience of end of life by caregivers (EOLE study)
Source: Ann Intensive Care. 2021 Nov 26;11:162. doi: 10.1186/s13613-021-00944-z (PMC8626545; doi:10.1186/s13613-021-00944-z)
Supplement: Supplementary file 1 — Additional file 1. Physician and Nurse CAESAR scores for patients with decision of carewithholding/withdrawal (N = 403). [file 13613_2021_944_MOESM1_ESM.pdf]

e Supplement 1: Physician and Nurse CAESAR scores for patients with decision of care withholding/withdrawal (N= 403)

Items for physicians and nurses		
	**Physician**<br>Mean score (±SD)	**Nurse**<br>Mean score (±SD)
**1. Was an EOL palliative care approach clearly decided for the patient?**<br>Please rate this experience: 1 Traumatic. 2 Painful. 3 Difficult. 4 Acceptable. 5 Comforting	4.32 (±0.57)<br>N=385	4.11 (±0.64)<br>N=379
**2. Was the decision to withhold or withdraw treatment clearly documented in the medical report?**<br>Please rate this experience: 1 Traumatic. 2 Painful. 3 Difficult. 4 Acceptable. 5 Comforting	4.33 (±0.58)<br>N=369	4.09 (±0.63)<br>N=313
**3. Do you think the patient received excessive or futile care?**<br>Please rate this experience: 1 Traumatic. 2 Painful. 3 Difficult. 4 Acceptable. 5 Comforting	4.19 (±0.6)<br>N=386	3.83 (±0.63)<br>N=379
**4. Was the patient able to communicate with you during his/her ICU stay?**<br>Please rate this experience: 1 Traumatic. 2 Painful. 3 Difficult. 4 Acceptable. 5 Comforting	3.94 (±0.57)<br>N=384	4.26 (±0.71)<br>N=380
**5. Was the patient's pain under control?**<br>Please rate this experience: 1 Traumatic. 2 Painful. 3 Difficult. 4 Acceptable. 5 Comforting	4.21 (±0.57)<br>N=378	3.72 (±0.76)<br>N=375
**6. Was the patient able to breathe comfortably?**<br>Please rate this experience: 1 Traumatic. 2 Painful. 3 Difficult. 4 Acceptable. 5 Comforting	3.93 (±0.61)<br>N=385	4.24 (±0.68)<br>N=381
**7. In your opinion. was the patient's dignity respected?**<br>Please rate this experience: 1 Traumatic. 2 Painful. 3 Difficult. 4 Acceptable. 5 Comforting	4.32 (±0.59)<br>N=386	4.2 (±0.77)<br>N=378
**8. Did the relatives pay regular visits to the patient?**<br>Please rate this experience: 1 Traumatic. 2 Painful. 3 Difficult. 4 Acceptable. 5 Comforting	4.27 (±0.67)<br>N=387	4.23 (±0.8)<br>N=366
**9. Did the ICU team discuss the patient's EOL wishes with the patient him/herself or with the relatives?**<br>Please rate this experience: 1 Traumatic. 2 Painful. 3 Difficult. 4 Acceptable. 5 Comforting	4.31 (±0.73)<br>N=385	3.91 (±0.82)<br>N=363
**10. Were the relatives at the patient's bedside at the time of death?**<br>Please rate this experience: 1 Traumatic. 2 Painful. 3 Difficult. 4 Acceptable. 5 Comforting	4.12 (±0.6)<br>N=389	4.03 (±0.91)<br>N=380
**11. During the patient's ICU stay. did the relatives receive support from a psychologist?**<br>Please rate this experience: 1 Traumatic. 2 Painful. 3 Difficult. 4 Acceptable. 5 Comforting	4.24 (±0.68)<br>N=388	3.85 (±0.7)<br>N=384
**12. Are you satisfied with the patient's overall quality of dying and death?**<br>Please rate this experience: 1 Traumatic. 2 Painful. 3 Difficult. 4 Acceptable. 5 Comforting	3.77 (±0.66)<br>N=383	3.76 (±0.75)<br>N=355
**13. If the patient had been your relative. would you have been satisfied with his/her EOL?**<br>Please rate this experience: 1 Traumatic. 2 Painful. 3 Difficult. 4 Acceptable. 5 Comforting	4.12 (±0.57)<br>N=388	4.02 (±0.71)<br>N=384
**Specific physician items**		
**14. Were the relatives able to say good-bye to the patient?**<br>Please rate this experience: 1 Traumatic. 2 Painful. 3 Difficult. 4 Acceptable. 5 Comforting	4.32 (±0.62)<br>N=388	
**15. Did you experience conflict with the patient and/or the relatives?**<br>Please rate this experience: 1 Traumatic. 2 Painful. 3 Difficult. 4 Acceptable. 5 Comforting	3.89 (±0.95)<br>N=387	
**Specific nurse items**		
**14. Were the relatives able to have physical contact (touch. hug) with the patient?**<br>Please rate this experience: 1 Traumatic. 2 Painful. 3 Difficult. 4 Acceptable. 5 Comforting	4.12 (±0.66)<br>N=383	
**15. Were you present at the patient's bedside at the time of death?**<br>Please rate this experience: 1 Traumatic. 2 Painful. 3 Difficult. 4 Acceptable. 5 Comforting	3.4 (±1.14)<br>N=373	
