# Supplementary material for: End of life in the critically ill patient: evaluation of experience of end of life by caregivers (EOLE study)
Source: Ann Intensive Care. 2021 Nov 26;11:162. doi: 10.1186/s13613-021-00944-z (PMC8626545; doi:10.1186/s13613-021-00944-z)
Supplement: Supplementary file 2 — Additional file 2. Physician and Nurse CAESAR scores for patients without decision of carewithholding/withdrawal (N = 107). [file 13613_2021_944_MOESM2_ESM.pdf]

e Supplement 2: Physician and Nurse CAESAR scores for patients without decision of care withholding/withdrawal (N= 107)

Items for physicians and nurses		
	**Physician**<br>Mean score (±SD)	**Nurse**<br>Mean score (±SD)
**1. Was an EOL palliative care approach clearly decided for the patient?**<br>Please rate this experience: 1 Traumatic. 2 Painful. 3 Difficult. 4 Acceptable. 5 Comforting	3.94 (±0.49)<br>N=98	3.8 (±0.67)<br>N=85
**2. Was the decision to withhold or withdraw treatment clearly documented in the medical report?**<br>Please rate this experience: 1 Traumatic. 2 Painful. 3 Difficult. 4 Acceptable. 5 Comforting	4.02 (±0.46)<br>N=54	3.8 (±0.68)<br>N=49
**3. Do you think the patient received excessive or futile care?**<br>Please rate this experience: 1 Traumatic. 2 Painful. 3 Difficult. 4 Acceptable. 5 Comforting	4.12 (±0.55)<br>N=102	3.97 (±0.58)<br>N=88
**4. Was the patient able to communicate with you during his/her ICU stay?**<br>Please rate this experience: 1 Traumatic. 2 Painful. 3 Difficult. 4 Acceptable. 5 Comforting	3.95 (±0.63)<br>N=100	4.27 (±0.67)<br>N=90
**5. Was the patient's pain under control?**<br>Please rate this experience: 1 Traumatic. 2 Painful. 3 Difficult. 4 Acceptable. 5 Comforting	4.32 (±0.55)<br>N=102	3.98 (±0.8)<br>N=91
**6. Was the patient able to breathe comfortably?**<br>Please rate this experience: 1 Traumatic. 2 Painful. 3 Difficult. 4 Acceptable. 5 Comforting	3.99 (±0.64)<br>N=101	4.22 (±0.59)<br>N=92
**7. In your opinion. was the patient's dignity respected?**<br>Please rate this experience: 1 Traumatic. 2 Painful. 3 Difficult. 4 Acceptable. 5 Comforting	4.28 (±0.49)<br>N=103	4.09 (±0.73)<br>N=86
**8. Did the relatives pay regular visits to the patient?**<br>Please rate this experience: 1 Traumatic. 2 Painful. 3 Difficult. 4 Acceptable. 5 Comforting	4.25 (±0.57)<br>N=103	4.15 (±0.68)<br>N=85
**9. Did the ICU team discuss the patient's EOL wishes with the patient him/herself or with the relatives?**<br>Please rate this experience: 1 Traumatic. 2 Painful. 3 Difficult. 4 Acceptable. 5 Comforting	3.89 (±0.92)<br>N=102	3.67 (±0.85)<br>N=86
**10. Were the relatives at the patient's bedside at the time of death?**<br>Please rate this experience: 1 Traumatic. 2 Painful. 3 Difficult. 4 Acceptable. 5 Comforting	3.96 (±0.52)<br>N=98	3.98 (±0.9)<br>N=88
**11. During the patient's ICU stay. did the relatives receive support from a psychologist?**<br>Please rate this experience: 1 Traumatic. 2 Painful. 3 Difficult. 4 Acceptable. 5 Comforting	4.01 (±0.86)<br>N=100	3.79 (±0.8)<br>N=91
**12. Are you satisfied with the patient's overall quality of dying and death?**<br>Please rate this experience: 1 Traumatic. 2 Painful. 3 Difficult. 4 Acceptable. 5 Comforting	3.66 (±0.71)<br>N=101	3.65 (±0.86)<br>N=86
**13. If the patient had been your relative. would you have been satisfied with his/her EOL?**<br>Please rate this experience: 1 Traumatic. 2 Painful. 3 Difficult. 4 Acceptable. 5 Comforting	3.99 (±0.52)<br>N=101	3.91 (±0.7)<br>N=90
**Specific physician items**		
**14. Were the relatives able to say good-bye to the patient?**<br>Please rate this experience: 1 Traumatic. 2 Painful. 3 Difficult. 4 Acceptable. 5 Comforting	4.22 (±0.64)<br>N=101	
**15. Did you experience conflict with the patient and/or the relatives?**<br>Please rate this experience: 1 Traumatic. 2 Painful. 3 Difficult. 4 Acceptable. 5 Comforting	3.75 (±0.84)<br>N=101	
**Specific nurse items**		
**14. Were the relatives able to have physical contact (touch. hug) with the patient?**<br>Please rate this experience: 1 Traumatic. 2 Painful. 3 Difficult. 4 Acceptable. 5 Comforting	3.89 (±0.84)<br>N=88	
**15. Were you present at the patient's bedside at the time of death?**<br>Please rate this experience: 1 Traumatic. 2 Painful. 3 Difficult. 4 Acceptable. 5 Comforting	3.33 (±1.11)<br>N=86	
