# Supplementary material for: End of life in the critically ill patient: evaluation of experience of end of life by caregivers (EOLE study)
Source: Ann Intensive Care. 2021 Nov 26;11:162. doi: 10.1186/s13613-021-00944-z (PMC8626545; doi:10.1186/s13613-021-00944-z)
Supplement: Supplementary file 3 — Additional file 3. Factors associated with CAESAR score (univariable analysis, comparisonbetween presence and absence of each variable, p significant when ≤ 0.05). [file 13613_2021_944_MOESM3_ESM.pdf]

e Supplement 3. Factors associated with CAESAR score (univariable analysis, comparison between presence and absence of each variable, p significant when ≤ 0.05).

		**Physicians' CAESAR Score** Mean ± SD	**P**	**Nurses' CAESAR Score** Mean ± SD	**P**
**During the stay in ICU N=510 patients**					
Vasoactive drugs	No N = 120	63 ±6	0.13	55 ±7	0.18
	Yes N = 390	62 ±5		56 ±5	
Invasive ventilation	No N = 75	63 ±6	0.98	53 ±7	<0.001
	Yes N = 435	63 ±5		56 ±5	
Disagreements between caregivers	No	62 ±5 N = 451	0.19	56 ±6 N = 421	<0.001
	Yes	61 ±6 N = 45		50 ±5 N = 59	
Disagreements between caregivers and relative	No	62 ±5 N = 458	0.73	55 ±6 N = 442	0.05

	Yes	60 ±7<br>N = 38		52 ±5<br>N = 34	
Disagreements between ICU caregivers and other department's physicians	No<br><br>Yes	62 ±5<br>N = 468<br>62 ±6<br>N = 25	0.39	55 ±6<br>N = 460<br>51 ±6<br>N = 14	0.02
Presence of nurse during the decision meeting	No<br><br>Yes	62 ± 4<br>N= 267<br>63 ± 5<br>N= 243	0.02	55 ± 6<br>N = 316<br>56 ± 5<br>N = 194	0.23
**The day of death**<br>**N=510 patients**					
Vasoactive drugs	No N=255<br><br>Yes N=256	62 ±6<br><br>62 ±5	0.26	56 ±6<br><br>55 ±6	0.14
Invasive ventilation	No N= 148<br><br>Yes N=362	62 ±6<br><br>62 ±5	0.41	55 ±7<br><br>56 ±5	0.23
Non-invasive ventilation	No N= 459<br><br>Yes N=50	62 ±5<br><br>61 ±6	0.35	56 ±6<br><br>53 ±7	0.02
Renal replacement therapy	No N=426	62 ±5	0.03	56 ±6	<0.001

	Yes N=76	61 ±5		52 ±6	
Cardiopulmonary Resuscitation	No N= 471	62 ±5	<0.001	56 ±6	0.01
	Yes N= 39	58 ±5		51 ±6	
Extra Corporeal Life Support	No N=489	62 ±5	0.94	55 ±6	0.61
	Yes N=21	62 ±3		54 ±7	
**After effective care withdrawal N=362 patients**					
Monitoring, blood tests	No N=329	63 ±5	0.04	57 ±5	0.02
	Yes N=30	62 ±5		55 ±7	
Immediate extubation	No N=174	63 ±5	0.98	55 ±6	0.04
	Yes N=87	63 ±5		57 ±5	
Sedation	No N=81	62 ± DS	0.36	55 ± 6	0.16
	Yes N=240	63 ± DS		56 ±5	
Delay of death > 1 day	No N=340	63 ±5	0.64	56 ±6	1.00

	Yes N=21	63 ±6		56 ±6	
